# Hyperspectral and genome-wide association analyses of leaf phosphorus status in local Thai indica rice

**DOI:** 10.1371/journal.pone.0267304

**Published:** 2022-04-20

**Authors:** Sompop Pinit, Natthanan Ruengchaijatuporn, Sira Sriswasdi, Teerapong Buaboocha, Supachitra Chadchawan, Juthamas Chaiwanon

**Affiliations:** 1 Faculty of Science, Department of Botany, Center of Excellence in Environment and Plant Physiology, Chulalongkorn University, Bangkok, Thailand; 2 Faculty of Science, Program in Biotechnology, Chulalongkorn University, Bangkok, Thailand; 3 Faculty of Medicine, Computational Molecular Biology Group, Chulalongkorn University, Bangkok, Thailand; 4 Faculty of Medicine, Research Affairs, Chulalongkorn University, Bangkok, Thailand; 5 Faculty of Science, Omics Sciences and Bioinformatics Center, Chulalongkorn University, Bangkok, Thailand; 6 Faculty of Science, Department of Biochemistry, Molecular Crop Research Unit, Chulalongkorn University, Bangkok, Thailand; Government College University Faisalabad, PAKISTAN

## Abstract

Phosphorus (P) is an essential mineral nutrient and one of the key factors determining crop productivity. P-deficient plants exhibit visual leaf symptoms, including chlorosis, and alter spectral reflectance properties. In this study, we evaluated leaf inorganic phosphate (Pi) contents, plant growth and reflectance spectra (420–790 nm) of 172 Thai rice landrace varieties grown hydroponically under three different P supplies (overly sufficient, mildly deficient and severely deficient conditions). We reported correlations between Pi contents and reflectance ratios computed from two wavebands in the range of near infrared (720–790 nm) and visible energy (green-yellow and red edge) (*r* > 0.69) in Pi-deficient leaves. Artificial neural network models were also developed which could classify P deficiency levels with 85.60% accuracy and predict Pi content with *R*^*2*^ of 0.53, as well as highlight important waveband sections. Using 217 reflectance ratio indices to perform genome-wide association study (GWAS) with 113,114 SNPs, we identified 11 loci associated with the spectral reflectance traits, some of which were also associated with the leaf Pi content trait. Hyperspectral measurement offers a promising non-destructive approach to predict plant P status and screen large germplasm for varieties with high P use efficiency.

## Introduction

Phosphorus (P) is an essential macronutrient that is critical for plant growth and development and crop productivity. Unlike nitrogen that can be acquired from N_2_ in the atmosphere through biological nitrogen fixation or the Haber-Bosch process, the global phosphorus resource is non-renewable. Global P supply from inorganic phosphate (Pi) rock reserves may be depleted within the next decades [1]. In spite of the high use of synthetic P fertilizers in agriculture, more than 80% of the supplied P is not used by crop plants [2]. P is often precipitated with other minerals or bound to organic compounds in the soil, while soil erosion is reported to account for 50% of total P losses [1], further aggravating the problem of P scarcity, as well as food security.

Plants have evolved a diverse array of adaptive mechanisms to respond to P deficiency. This includes remodeling of root system architecture to explore more soil volumes and secreting root exudates, such as acid phosphatases or organic acids, to enhance P acquisition efficiency [3]. P-deficient plants can also recycle internal Pi or alter P remobilization from mature to young developing organs to improve internal P use efficiency [4]. Identification of germplasm with high P use efficiency and genes underlying the mechanism could lead to the development of highly P-efficient crops that can tolerate P deficiency stress and maintain productivity.

Hyperspectral technology uses visible (VIS, 400–700 nm), near infrared (NIR, 700–1100 nm) and shortwave infrared (SWIR, 1100–2500 nm) energy to estimate plant physiological and biochemical properties. This technique offers a promising tool to quickly and non-destructively phenotype plant leaves. Photosynthetic pigments in leaf cells absorb most of the spectra in VIS, while water in the leaves reflects NIR and absorbs certain wavebands in SWIR [5]. Thus, variability in pigment compositions and concentrations of water, inorganic minerals or organic compounds in plant tissues could lead to different spectral reflectance. Based on these properties, narrow-band vegetation indices, which are computed from two or more spectral bands in a simple mathematical form, have been investigated for their performance on predicting various leaf traits, as determined by correlation analysis. Notably, the normalized difference vegetation index (NDVI) is widely used to estimate leaf chlorophyll and nitrogen levels [6].

The advanced development of massively parallel sequencing technologies has enabled researchers to perform genome-wide sequencing analyses and explore allelic diversity existing in populations. Genome-wide association study (GWAS) has become an effective method for dissecting the genetic basis of the complex traits by establishing statistical links between phenotypes and genotypes [7]. As such, there is an increasing need to phenotype plants or screen large collections of germplasm to search for accessions with desired phenotypes.

In Thailand, soil P contents are very low [8]. With the long history of rice cultivation, Thai rice landrace accessions may potentially adapt to the low P soil conditions. In this study, we evaluated the relationship between leaf Pi content, shoot biomass, and leaf spectral reflectance from a rice panel consisting of 172 Thai landrace accessions grown hydroponically in three different P supplies. The hyperspectral data (380–790 nm) were measured non-destructively with a handheld spectrometer, and leaf samples were harvested for laboratory Pi content determination. Classification of P status and prediction of Pi content using deep learning models, as well as regression analysis, were performed. The measured Pi contents and reflectance indices computed from two wavebands were used to perform GWAS analysis to identify genetic loci related to P efficiency in rice.

## Materials and methods

### Plant materials and growth conditions

Seeds of 172 local Thai rice (*Oryza sativa* L. subsp. *indica*) accessions (S1 File) were provided by the Pathum Thani Rice Research Center and grown in hydroponic conditions. Seeds were sterilized using commercial bleach (2% sodium hypochlorite), germinated in water for 2 days, and then pre-cultivated in half-strength Yoshida’s solution [9] for 5 days. Then, the seed endosperm was removed from the seedlings, and the seedlings were transferred to full-strength Yoshida’s solution with 3 different levels of P concentrations (320, 16 and 0.8 μM NaH_2_PO_4_ for P100, P5 and P0.25 treatments, respectively) and maintained for 16 days. The decrease of NaH_2_PO_4_ of each treatment was supplied with NaCl to reach the same concentration of Na^+^ in the control (full-strength Yoshida’s solution).

The experiment was performed in a randomized complete block design (RCBD) and repeated three times. Each experiment included three plants/accession/treatment. Seedlings were grown in 80-litre containers, each containing all 172 accessions (one plant per accession). The nutrient solution was renewed every 4 days and adjusted pH at 5.8 every two days. The experiment was performed in the greenhouse under natural light conditions (30–38°C day/26-30°C night temperature; 40–70% day/70-90% night relative humidity). The plants were harvested after 16 days of treatment. The second fully expanded leaves were used for leaf spectral reflectance measurement, and leaf discs were harvested for Pi content determination (see below). The presence of leaf senescence (with at least half of the leaf blade showing senescence) in the first three leaves was recorded. The remaining shoot samples were harvested, dried in oven at 80°C for 3 days and used for biomass (dry weight) measurement. Ten accessions were randomly selected for total root length measurement.

### Pi extraction and determination

After spectral reflectance measurement (see below), the same leaves were harvested for Pi content determination using the Pi-molybdenum blue assay described previously [10]. In brief, each leaf was punched using a paper puncher to harvest four 3-mm-diameter leaf discs. The leaf discs were immediately put into a 96-well plate on dry ice and then stored at -80°C. Leaf Pi was extracted by incubating the leaf discs in 5.5% (w/v) perchloric acid for 3 hours. The Pi concentration in the supernatants was then measured using the molybdate blue assay. Absorbance was measured at 820 nm using a “SpectraMax M3” microplate reader (Molecular Devices, USA). A standard curve was performed using different concentrations of KH_2_PO_4._ The Pi concentration was calculated by comparing A_820_ with the standard curve and was calculated as nmol per leaf area (mm^2^).

Phosphorus Utilization Efficiency (PUtE) was calculated as the ratio of shoot biomass divided by the Pi content measured from the same plant [11]. Hierarchical clustering of the variances of mean Pi contents, biomass, and PUtE of different rice varieties was performed and plotted using the ClustVis web tool [12].

### Spectral reflectance measurement and correlation with Pi content

Leaf spectral reflectance (*R*_λ_: the reflectance at respective wavelengths, λ) was measured using a handheld spectroreflectometer (PolyPen RP 400 (UV-VIS), Photon Systems Instruments, Brno, Czech Republic), which scanned wavebands from 380 to 790 nm in 2 nm steps. The device was calibrated with a white reference standard before use. For each leaf, the measurements were performed twice at two points near the center of the leaf length by placing the adaxial side to face PolyPen’s measuring head. Spectral reflectance data were exported from the device and further analyzed in Excel. The two measurements from the same leaf were treated as technical replicates, and the values were averaged. Then, means of *R*_λ_ for each wavelength from all rice accessions were calculated for different P treatments. Spearman’s Rank correlations between *R*_λ_ from two different wavelengths and between reflectance ratio (*R*_NIR_ /*R*_VIS_) and Pi contents were calculated. Heatmaps were plotted using the ClustVis web tool [12].

To model the relationship between reflectance characteristic (e.g., *R*_λ_ or a reflectance ratio) and Pi content, regressions with exponential decay function, $\mathrm{Reflectance}=a+be^{-c∙\mathrm{Pi}}$, were performed. The exponential decay function was chosen because reflectance values tend to change sharply at low Pi content (Pi < 0.1 nmol/mm^2^) and flatten out over the intermediate Pi content range (0.2 < Pi < 0.5 nmol/mm^2^). Furthermore, we trimmed data points with Pi content below 0.02 nmol/mm^2^ where most measurements are expected to be noises. The value of the offset term *a* in the exponential decay function was constrained within the interval [−1, ∞), as reflectance values are positive. The value of the exponent term *c* was constrained within the interval [0, −∞) to preserve the exponential decay characteristic. The value of the multiplicative factor term *b* was unconstrained. The covariance matrices of the three regression parameters and the *R*-squared statistics (*R*^2^) were calculated to monitor the quality of the fit. Regressions were performed at both individual plant level and at accession level, where average values from plants in the same accession and P treatment were used.

Mean normalized values of reflectance ratio indices were calculated from the mean of each reflectance ratio of the P0.25 treatment divided by the corresponding reflectance ratio of the control (P100 treatment). Total of 217 mean normalized SR traits were used in GWAS.

### Deep learning model development

Leaf spectral reflectance data were standardized so that the reflectance at each wavelength has zero mean and unit variance prior to inputting into the model. The total of 6,258 reflectance spectra were split into 4,379 for training, 939 for validation, and 940 for testing. To increase the number of samples, synthetic reflectance spectra were additionally generated by adding a Gaussian noise with standard deviation of 0.01 to the observed spectra. These augmented samples were used only for training the models and excluded from performance evaluations.

Two model architectures were explored (Fig 2A): a standard convolutional neural network with a single classification output, and a multi-task model that includes both a classification output and a reconstructed spectra output. The spectral reconstruction objective was added to encourage the multi-task model to learn a meaningful low-dimensional representation of the input reflectance spectra. In both model variants, data from the input standardized spectra passed through two convolutional blocks, each consisting of a 1D convolutional layer, a batch normalization layer, and a rectified linear unit (ReLU) activation layer. The first block has 16 filters of kernel size 8 and the second block has 32 filters of kernel size 8. The output from the second block then passed through an average pooling layer with a window size of 4 and a stride of 3. Finally, the pooled output was flattened into a 1D vector (flattened output) and passed through two fully connected layers with hidden dimensions of 64 and 3, respectively. The last layer produces 3 outputs which are the predicted probabilities for P100, P5, and P0.25 conditions. Dropout layers with dropout rate of 0.2 were added before the fully connected layers as a regularization. The hidden dimension of the first fully connected layer was also varied from 32 to 1,024 to optimize the performance of the models.

For the multi-task model, the flattened output from the average pooling layer also passed through a fully connected layer with hidden dimension of 1,664 and then reshaped into a (52, 32)-dimensional tensor. This tensor then passed through two upsampling blocks, each consisting of a 1D convolutional layer, a batch normalization layer, a ReLU activation layer, and an upsampling layer with upsampling size of 2. The first block has 32 filters of kernel size 8 and the second block has 16 filters of kernel size 8. Finally, the upsampled output passed through a 1D convolutional layer with 1 filters of kernel size 4 to reconstruct the input spectrum.

Categorical cross-entropy was used as the loss function for the classification and the mean square error was used as the loss function for the reconstruction of input spectrum. The multi-task model combines the two losses with a weight of 1.0 for classification and 0.5 for spectral reconstruction. Model performances were measured from 5 random initializations. The categorical cross-entropy is defined as:

$$crossentropy\;loss=-\sum_{i}y_{i}\;log\;p_{i}$$

where *y*_*i*_ is 1 if the input spectrum of class *i*, and 0 otherwise and *p*_*i*_ is the predicted probability for class *i*. The reconstruction loss is defined as:

$$reconstruction\;loss=\sum_{i}\frac{1}{n}\sum_{j=1}^{n}{\left(s_{j}-{\hat{s}}_{j}\right)}^{2}$$

where n is the number of wavelengths, *s*_*j*_ is the observed value at wavelength *j*, and ${\hat{s}}_{j}$ is the predicted value for wavelength *j*.

For the prediction of log_10_ Pi content, the classification output head was replaced by a linear output head. Mean square error was used as the loss function for optimizing the regression performance. Absorbance spectra for 3,791 P5 and P0.25 plants were split into 2,651 for training, 571 for validation, and 569 for testing. All individual plants of the same accessions were assigned to the same set. Adding data from P100 plants to the training set significantly degrades the regression performance from an overall *R*^*2*^ of 0.53 to 0.38.

### Class activation map generation

To understand how the model makes use of the input spectrum, we applied the gradient-weighted class activation map (Grad-CAM) [https://arxiv.org/abs/1610.02391] which is a standard technique in computer vision for acquiring visual explanation of deep models. Grad-CAM produces a Class Activation Map (CAM) that identifies discriminative regions of an input for a given class. The first step in Grad-CAM is to compute the gradients of the signal for a given class with respect to the output of the last convolution block of the model. Conceptually, the average of these gradients reflects the importance of each input feature (or wavelength in this case) for the given class. Only the regions with a positive effect on the interested class are obtained. For the visualization of CAMs in Fig 2C, the averaged gradients were normalized by the sum within each sample.

### Association mapping

For each accession, means of the Pi contents, biomass, and PUtE under different P treatments (with the exception of PUtE at P100) and normalized reflectance ratios (SR traits) were calculated and used for association mapping. The whole-exome SNP data and population structure of 172 Thai rice accessions were obtained from a previously reported association mapping study [13]. SNPs with minor allele frequency (MAF) < 0.05 were filtered out. The remaining 113,114 SNPs were used for the association mapping with the Pi contents and SR traits. The GWAS analysis was performed using the linear mixed model (LMM) of GEMMA software [14]. Association results were illustrated with Manhattan plots and quantile–quantile (Q-Q) plots. The plots were generated using the ‘qqman’ package [15] in R (version 3.6.1; R Core Team, 2019). Significant SNPs were considered using Bonferroni correction with an experimental type I error rate of α = 0.05, which the significant threshold of these associations was -log_10_(*p*-value) ≥ 6.35.

Results of linkage disequilibrium (LD) analysis between pairs of SNPs were obtained from the previous study [13]. SNPs located within 100 kb of the significant SNPs and LD correlation *r*^2^ values greater than 0.50 were included for candidate gene analysis.

Gene models and their annotation were obtained from the MSU Rice Genome Annotation Project database [16]. For each significant SNP and LD block, gene models in the LD block were retrieved from the MSU database. Putative candidate genes were selected based on their characterized and annotated function by searching against the funRiceGenes dataset [17] and the MSU Rice Genome Annotation Project database [16].

### Statistical analysis of phenotypic data

The effects of genotype, treatment and their interaction were tested using two-way analysis of variance (ANOVA) in SPSS version 22. The distribution of of Pi content and biomass was visualized using the seaborn package v0.9.0 in Python 3.8. Phenotypic comparison between treatments was performed using ANOVA in SPSS version 22.

## Results

### Phenotypic variations of Pi contents and plant growth in different P supplies

**Fig 1:**
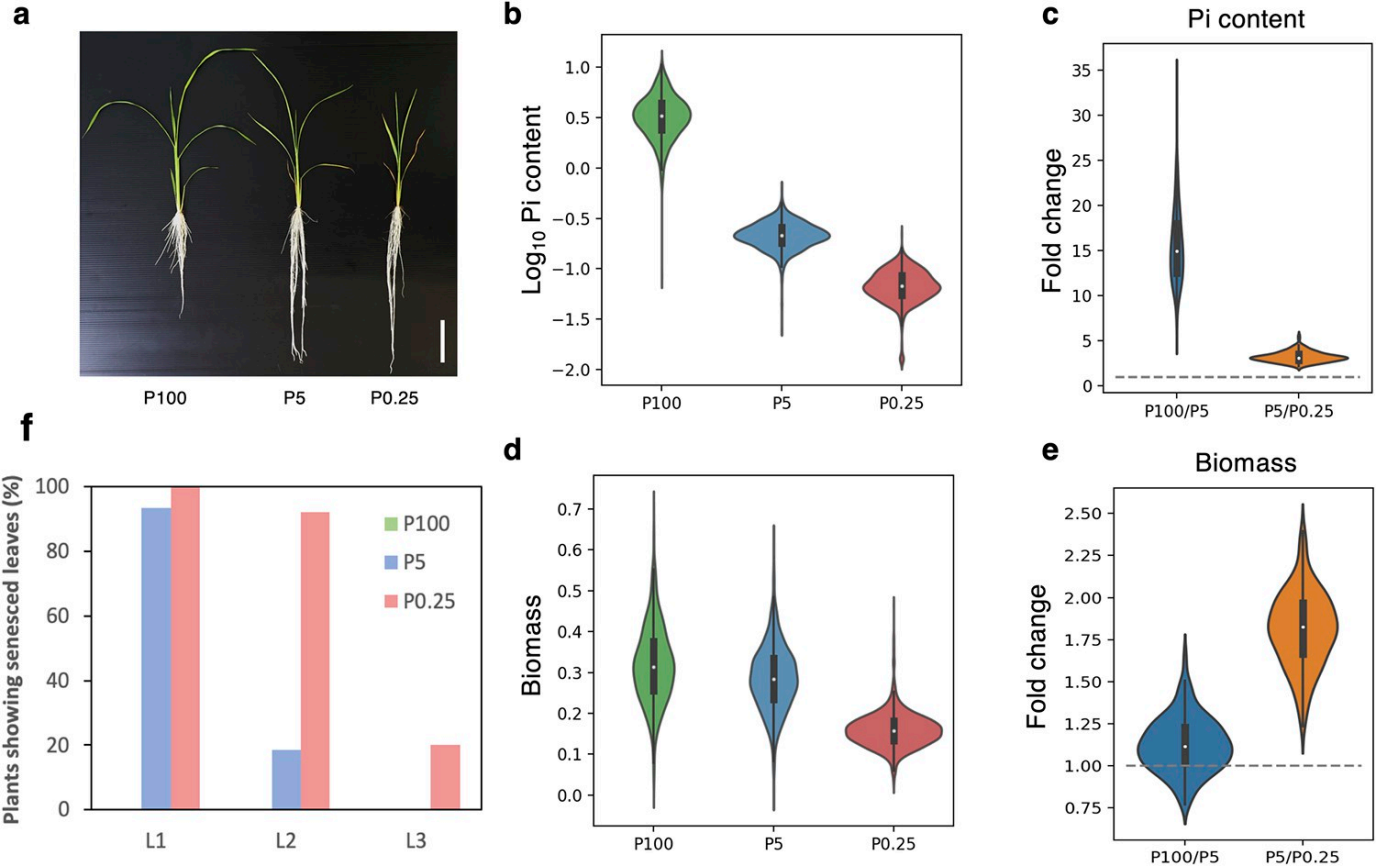
Phenotypes of rice seedlings grown under different P conditions. (a) A representative image of rice seedlings grown in P100, P5 and P0.25 conditions, showing plant size, root length and leaf senescence. Scale bar = 5 cm. (b, d) Frequency distribution of leaf Pi content (nmol/mm^2^) (b) and biomass (shoot dry weight, g/plant) (d). The values in the X axis of (b) are marked with both linear and logarithmic scales. (c, e) Fold change comparison of leaf Pi content (c) and biomass (e) in P100 vs. P5 and P5 vs. P0.25 conditions. (f) Percentage of plants showing senescence in the oldest (L1), the second oldest (L2) and the third oldest (L3) leave. For each P condition, the number of plants analyzed was at least 1,500 plants. P100, P5 and P0.25 conditions contained 320, 16 and 0.8 μM NaH_2_PO_4_, respectively.

To evaluate phenotypic variations in the rice panel and their responses to Pi deficiency, we determined Pi content and spectral reflectance (380–790 nm) in the second fully expanded leaves, as well as biomass (shoot dry weight) and chlorosis or senescence in older leaves, of 172 rice accessions grown in full-strength Yoshida’s nutrient solution with three different P supplies: 320, 16 and 0.8 μM P, denoted as P100, P5 and P0.25, respectively (Fig 1A).

Frequency distribution analysis of leaf Pi content exhibited a normal distribution pattern under all three P conditions (Fig 1B). The non-overlapping graphs of each condition indicated that the plants accumulated different levels of Pi as a result of the different P treatments. When P supplies were reduced from P5 to P0.25, all rice accessions showed statistically significant decreases in leaf Pi content and shoot biomass with an average of 3.23 and 1.81 fold decreases, respectively (Fig 1B–1E). On the other hand, when P supplies were reduced from P100 to P5, leaf Pi contents decreased by an average of 15.62 fold, but shoot biomass was only slightly reduced by 1.12 fold (Fig 1B–1E). Analysis of leaf senescence in the first three leaves showed that 93.3% of the oldest leaves and 18.5% of the second oldest leaves in P5 showed senescence, while none of the leaves in P100 showed senescence (Fig 1F). The presence of senesced leaves in P5, but not P100, suggests that the P5 level was suboptimal as plants responded to P deficiency by remobilizing P from mature leaves to the younger leaves. Furthermore, as compared to P100, the P5 treatment increased root lengths, indicating an adaptive response to P deficiency (Figs 1A and S1). These findings suggest that the P0.25, P5 and P100 treatments, respectively, represented severely deficient, mildly deficient, and overly sufficient conditions for rice growth in this study. Under P5 condition, the results showed that internal Pi contents from mature leaves were remobilized to younger leaves without significant growth retardation (Fig 1D and 1E). This suggests that the Pi contents of the second fully expanded P5 leaves could be used to study plant adaptation to P deficiency. However, the extra amount of Pi observed in the P100 leaves may not be relevant to normal plant growth.

To assess P deficiency adaptation across the Thai landrace varieties, we calculated P utilization efficiency (PUtE) by dividing shoot biomass by each plant’s Pi content [11]. The variances of mean Pi contents, biomass, and PUtE of each variety were clustered hierarchically. PUtE at P5 and P0.25 was more similar to biomass under the same conditions, however PUtE at P100 was not because the Pi content were extremely high and may not be physiologically relevant to plant growth (S2 Fig). As a result, PUtE at P100 was not included in the following association study. Clustering analysis of rice varieties identified those with (1) high Pi accumulation, low biomass and low PUtE, (2) low Pi accumulation, high biomass and high PUtE, (3) high Pi accumulation, high biomass and moderate PUtE, and (4) low Pi accumulation, low biomass and moderate PUtE, as well as other groups with varying Pi accumulation patterns under different P conditions (S2 Fig). These results suggest that the rice varieties in this panel exhibited varied patterns of Pi accumulation, growth and PUtE and used different adaptation strategies in response to limited P supply.

### Spectral reflectance characteristics of leaves with varying Pi contents

**Fig 2:**
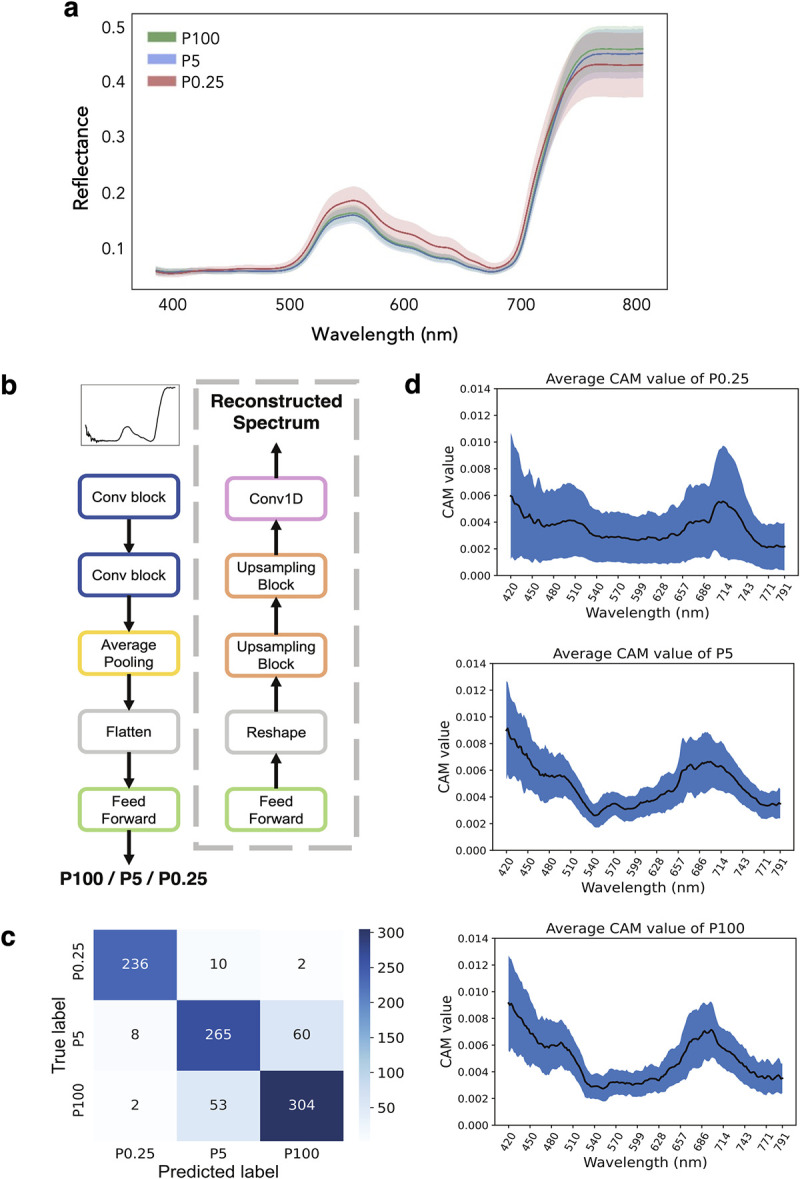
Spectral reflectance of the leaves from different P conditions and classification of P deficiency levels using artificial neural network model. (a) Average reflectance spectrum (380–790 nm). Shaded areas indicate the one-standard deviation range. (b) Overview of the model architectures for the standard convolutional neural network (outside of the dashed box) and the multi-task model (including the dashed box). See Methods for the detailed description of the architecture. (c) Confusion matrix of the best model (multi-task model with hidden dimension = 64) on the test set (*n* = 940). (d) Class activation maps (CAMs) for samples from each P deficiency class. CAM values conceptually represent the relative importance of the data from each wavelength on the predicted class (see Methods). Black trend lines indicate the running mean. Shaded areas indicate the one-standard deviation range.

Spectral reflectance measured from the second fully expanded leaves grown under different P treatments showed similar overall patterns with typical features of fresh plant leaves, including the low reflectance at the blue and red bands and the red edge [5]. Comparing the mean spectra of different P treatments, the results showed that P0.25 treatments increased the reflectance in the VIS region, likely due to reduction of chlorophyll, but decreased the reflectance in the NIR region (730–790 nm). P5 treatments slightly decreased the reflectance in the NIR region, but did not affect the reflectance in the VIS region (Fig 2A). Although the P5 plants had much lower Pi content than the P100 plants (Fig 1B and 1C), they did not show obvious visible deficiency symptoms in terms of leaf spectral reflectance or chlorosis in the second fully expanded leaves. This result indicates that spectral reflectance is affected by P deficiency when plants are severely P-deficient. However, leaf Pi contents reflect P status more accurately and are more sensitive to P deficiency than spectral reflectance when plants are under mildly deficient conditions.

The whole VIS-NIR spectra are rich in data. To explore the correlation structure of the wavebands, we first performed correlation analysis between reflectance at two wavelengths of each spectrum (within-spectrum) using spectral data from 172 rice accessions grown under different P conditions (S3A Fig). A correlation matrix heatmap illustrates that reflectances in the VIS range (~420–720 nm) and the NIR range (~730–780 nm) are highly correlated within each group (*r* = 0.53–0.99) but not across the two groups (*r* = 0.05–0.54) (S3A Fig). In contrast, data from 380–410 nm was highly fluctuating, showing low correlations between adjacent wavelengths (e.g., within 10 nm). This suggests that data from 380–410 nm was noisy and thus excluded from further consideration in this study.

### Artificial neural network models for detecting P deficiency

With more than 6,000 high-resolution leaf reflectance spectra generated, we trained artificial neural networks to predict plant P status (P100, P5, or P0.25). Two model variants were developed: a standard convolutional neural network (CNN) and a multi-task network with both a classification output and a spectral reconstruction output (Fig 2B). The spectral reconstruction output was added to encourage the model to learn meaningful low-dimensional representation of the input spectra. This helps the models generalize better to unseen input spectra (S1 Table). The best multi-task model achieved 85.60% classification accuracy, with the majority of errors occurring between P100 and P5 conditions (Fig 2C). The P0.25 condition can be accurately differentiated.

To understand how the artificial neural network models distinguish the reflectance spectra between the P100, P5, or P0.25 conditions, we generated a class activation map (CAM) for each correctly classified input. CAM illustrates the contribution of reflectance value from each wavelength on the predicted class through the artificial neural network architecture. This showed that the models consistently focused on the 680–700 nm and the 420–500 nm regions, especially for the P100 and P5 samples (Fig 2D). In contrast, CAMs for P0.25 samples are flatter and more highly variable. This may be because the reflectance spectra for P0.25 differ from the other classes on almost the entire waveband (Fig 2A), and so many parts of the spectra could be used to distinguish them.

### Correlation and regression analyses of Pi contents

Self-correlation analysis of the reflectance values indicated that the entire spectrum (380–790 nm) could be reduced to two regions: the VIS region (*R*_VIS_) and the NIR region (*R*_NIR_). As such, reflectance ratios computed from the two wavebands (*R*_NIR_ /*R*_VIS_) could be simple yet informative representations of the complex spectral data. In the following analyses, reflectance data at 10 nm intervals (e.g., *R*_420_, *R*_430_, …, *R*_790_) were considered for building ratios. In total, there are 217 reflectance ratios calculated from 7 *R*_NIR_ (*R*_730_, *R*_740_, …, *R*_790_) and 31 *R*_VIS_ (*R*_420_, *R*_430_, …, *R*_720_) values.

**Fig 3:**
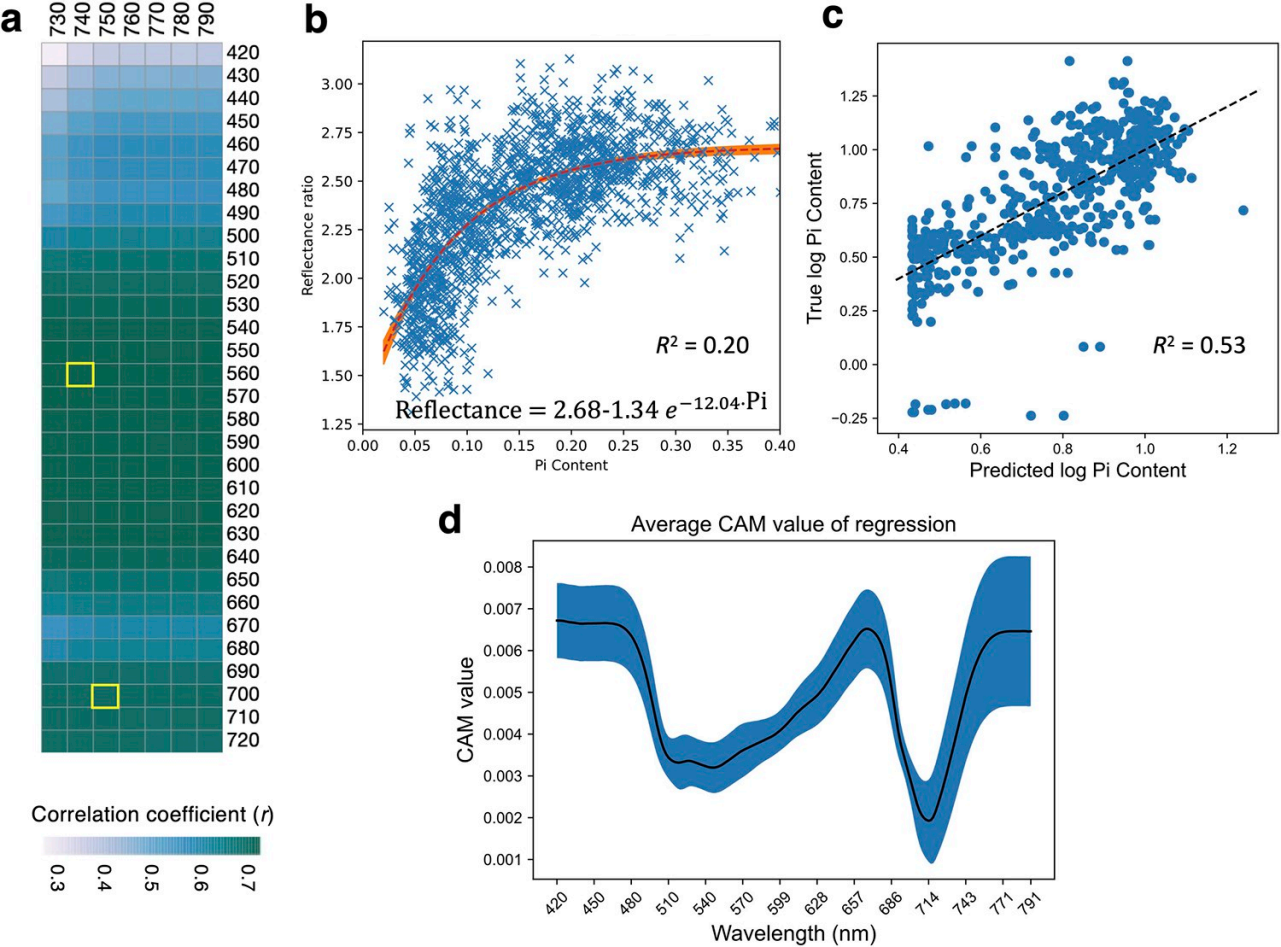
Correlation and regression analyses of Pi contents and reflectance ratio indices. (a) Heatmap shows Spearman’s correlation between 217 reflectance ratio indices (*R*_NIR_ /*R*_VIS_) and leaf Pi content determined from the same leaves. *R*_740_/*R*_560_ (*r* = 0.72) and *R*_750_/*R*_700_ (*r* = 0.70) (marked with yellow squares) were selected for a regression analysis with Pi contents (nmol/mm^2^) (b). Pi content and *R*_750_/*R*_700_ data was fitted by a non-linear regression model with an exponential decay function. The formula and *R*^*2*^ statistics are displayed in the graph. Each data point is from an individual plant (*n* = 172 accessions x 2 P treatments (P5 and P0.25) x 3 individual plants x 3 independent experiments). (c) Scatter plot showing the correlation between observed Pi contents and the predicted values made by an artificial neural network model on a held-out test set consisting of 569 plants. The corresponding *R*^*2*^ is 0.53. (d) Class activation map (CAM) showing the relative importance of the data from each wavelength on the predicted Pi content (see Methods). Black trend lines indicate the running mean. Shaded areas indicate the one-standard deviation range.

To determine VIS-NIR reflectance indices that are relevant to leaf Pi contents and growth parameters, we performed Spearman’s correlation analysis between each of the 217 reflectance indices and leaf Pi contents, shoot biomass and PUtE determined from the same leaves. The data was taken from all 172 rice accessions grown in the P5 and P0.25 treatments. Correlation matrix heatmaps showed that the indices with *R*_NIR_ from 730–790 nm and *R*_VIS_ from 530–630 nm or 700–720 nm, showed strong correlation with Pi content (*r* > 0.69) and good correlation with shoot biomass (*r* > 0.55) and PUtE (*r* < -0.47), all of which exhibited similar correlation patterns (Figs 3A and S3B). These results suggest that reflectance indices computed from these indices may be used to predict leaf Pi contents as well as growth-related parameters in rice.

We selected one of the best correlated reflectance indices from each region, which included *R*_740_/*R*_560_ (*r* = 0.72) and *R*_750_/*R*_700_ (*r* = 0.70), and performed a regression analysis with Pi contents. The data was fitted by a non-linear regression model with an exponential decay function, as the reflectance ratios changed dramatically when Pi contents were less than 0.1 nmol/mm^2^ but changed slowly when Pi contents were between 0.2–0.4 nmol/mm^2^ (Figs 3B and S4). When data points from individual plants were considered, the regression achieved *R*^*2*^ of 0.20 for *R*_740_/*R*_560_ and *R*_750_/*R*_700_ (Figs 3B and S4A). When mean values of each rice accession (*n* = 9–12 plants per accession) were used, the regression achieved higher *R*^2^ of 0.52 and 0.69 for *R*_740_/*R*_560_ and *R*_750_/*R*_700_ (S4b and S4c Fig), respectively, with less than 10% coefficient of variations in the estimated parameter values (S4B and S4C Fig).

Next, the same artificial neural network architectures used for predicting plant P status (Fig 2B) were adapted for predicting log_10_ Pi content by replacing the 3-class output with a single linear output. This significantly improved the *R*^*2*^ of Pi content regression from 0.20 to 0.53 for individual plants (Fig 3C). The multi-task model with spectral reconstruction achieved the best performance with mean absolute error of 0.14 on both P5 and P0.25 plants. The class activation map, which shows the relative importance of each wavelength on the model output, indicated that the 420–500 nm, 660–670 nm, and the 760–790 nm regions were most important (Fig 3D).

### Genome-wide association analysis using Pi contents and spectral reflectance traits

Statistical analysis of the mean Pi content, biomass, PUtE and the two selected reflectance ratio (*R*_740_/*R*_560_ and *R*_750_/*R*_700_) values in different P treatments showed considerable phenotypic variation within the accession panel, as indicated by the coefficient of variation (CV) (6.20–48.40%) (Table 1). Effects of the genotype, treatment, and genotype x treatment interaction on the observed phenotypic data were also significant (S2 Table), indicating that the phenotypic data could be used for association mapping.

**Table 1:** Descriptive statistics of Pi content, shoot biomass, PUtE and reflectance ratio phenotypic values from 172 accessions.

Traits	Treatments	Mean±SD	Range	CV (%)
**Pi content** **(nmol/mm^2^)**	P100	3.60 ± 1.74	0.08–11.56	48.40
	P5	0.22 ± 0.07	0.03–1.00	30.89
	P0.25	0.07 ± 0.03	0.01–0.22	35.00
**Shoot biomass** **(g)**	P100	0.32 ± 0.06	0.11–0.46	19.55
	P5	0.29 ± 0.05	0.13–0.47	18.93
	P0.25	0.16 ± 0.03	0.06–0.27	18.78
**PUtE^a^** **(g Biomass/**	P100 P5	0.04 ± 0.01 0.47 ± 0.12	0.01–0.08 0.13–0.84	29.11 24.68
**mg/g Pi content)**	P0.25	0.85 ± 0.20	0.22–1.45	23.13
***R*_740_/*R*_560_**	P100	2.95 ± 0.18	1.13–3.58	6.20
	P5	2.97 ± 0.21	2.28–3.72	7.15
	P0.25	2.43 ± 0.29	1.71–3.50	11.92
***R*_750_/*R*_700_**	P100	2.53 ± 0.17	1.02–3.10	6.86
	P5	2.56 ± 0.19	1.91–3.13	7.44
	P0.25	2.12 ± 0.29	1.31–2.97	13.68

^a^ Pi content used in the calculation was converted from the unit of nmol/mm^2^ to mg/g by multiplying with the molecular weight of PO_4_^3-^ and dividing with dry weight of leaf discs with known leaf areas.

**Fig 4:**
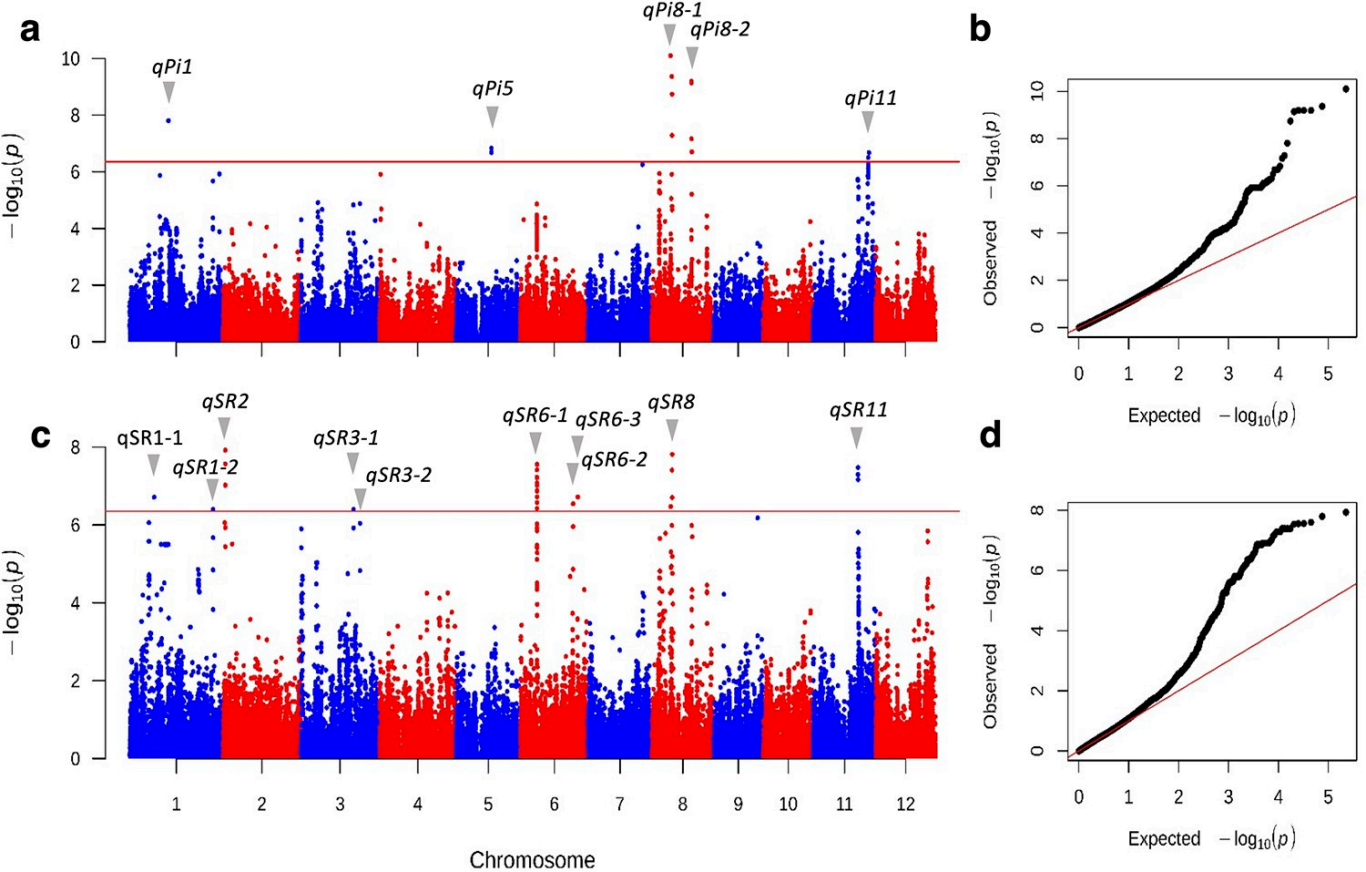
Manhattan plots and quantile-quantile plots from GWAS of leaf Pi content and reflectance ratios. (a) Manhattan plot for Pi_P5 (Pi content determined from the P5 treatment). (b) QQ plot for Pi_P5. (c) Manhattan plot for *R*_750_/*R*_700_ index of P0.25 treatment normalized by P100 treatment (d) QQ plot for the *R*_750_/*R*_700_ index. For Manhattan plots, the x-axis represents SNP positions across the entire rice genome by chromosome, and the y-axis is the -log_10_(*p*-value) of each SNP. Red lines indicate the threshold line at -log_10_(*p*-value) ≥ 6.35.

To identify genes associated with P deficiency responses in the Thai rice population, we performed a genome-wide association study (GWAS) using published exome-sequencing SNP data [13], which included 172 accessions used in this study. After filtering out SNPs with minor allele frequency (MAF) < 0.05, the remaining 113,114 SNPs were used for association mapping with the phenotypic data using the linear mixed model (LMM) of GEMMA software [14]. SNPs that passed a significant threshold of -log_10_(*p*-value) ≥ 6.35 were considered significant SNPs. For each trait, significant SNPs located within an approximately 300-kb region were considered as one association signal (locus), and SNPs with the lowest *p*-value were considered as lead SNPs. For mean Pi content under different P treatments (denoted as Pi_P100, Pi_P5 and Pi_P0.25), we identified 5 loci associated with the Pi_P5 trait (Fig 4A and 4B). The details about these loci are listed in Table 2. There was no significant SNP found in the Pi_P100 and Pi_P0.25 trait, as well as the PUtE_P5 and PUtE_P0.25 trait. (S5 Fig). The PUtE_ P100 trait was not included in this analysis because leaf Pi content was extremely high and not relevant to its biomass at P100 condition.

**Table 2:** List of loci associated with Pi content and spectral reflectance indices. The number of significant SNPs and -log_10_ (*p*-value) for each locus of the reflectance ratios *R*_740_/*R*_560_ and *R*_750_/*R*_700_ are listed and separated by semicolons.

Trait	Loci name	Chr	Number of significant SNPs	Lead SNP position (bp)	-log_10_ (*p*-value)	Minor allele frequency
**Pi_P5**	*qPi1*	1	1	18,062,446	7.81	0.101
	*qPi5*	5	2	16,423,976	6.83	0.057
	*qPi8-1*	8	4	8,596,112	10.11	0.052
	*qPi8-2*	8	6	18,346,725	9.19	0.054
	*qPi11*	11	2	25,844,888	6.68	0.054
**SR (*R***_**740**_ **/*R***_**560**_ **; *R***_**750**_ **/*R***_**700**_ **)**	*qSR1-1*	1	0; 2	11,321,950	6.71	0.06
	*qSR1-2*	1	0; 1	38,610,955	6.40	0.114
	*qSR2*	2	5; 4	1,002,248	7.94; 7.92	0.075
	*qSR3-1*	3	3; 1	24,458,607	6.69; 6.40	0.106
	*qSR3-2*	3	1; 0	27,448,860	6.39	0.062
	*qSR6-1*	6	29; 30	7,583,291	7.60; 7.41	0.194
	*qSR6-2*	6	0; 2	24,291,698	6.54	0.135
	*qSR6-3*	6	1; 1	26,513,707	6.77; 6.72	0.054
	*qSR8*	8	4; 4	9,225,490	7.39; 7.41	0.085
	*qSR11*	11	5; 3	20,701,489	7.56; 7.47	0.062

To determine genetic loci associated with plant growth and spectral reflectance (SR) in response to mild and severe Pi deficiency, the phenotypic values of the P-deficient conditions (P5 and P0.25) were divided by those of the P100 condition in which plants were not stressed. The mean normalized values of biomass and 217 reflectance ratios computed from 7 *R*_NIR_ and 31 *R*_VIS_ (*R*_NIR_ /*R*_VIS_) were used for association mapping, which was carried out in the same way as for the Pi content and PUtE traits. Of the 217 reflectance ratios from P0.25 condition (P0.25/P100), 113 of them did not identify any significant SNP, while 104 reflectance ratios identified from 1 to 48 significant SNPs (S6 Fig). The lists of the significant SNPs from each SR trait are highly overlapped due to the strong correlations between the reflectance ratios and leaf Pi contents (Fig 3A). All of the significant SNPs identified from the SR traits can be summarized into 10 loci (Table 2). In particular, the trait *R*_750_/*R*_700_ identified 48 significant SNPs corresponding to 9 out of the 10 loci (Fig 4C and 4D), and the trait *R*_740_/*R*_560_ identified 48 significant SNPs corresponding to 7 out of the 10 loci. However, neither the *R*_750_/*R*_700_ nor the *R*_740_/*R*_560_ traits from the P5 condition identified significant SNPs, most likely due to the very small difference in reflectance spectra between the two conditions (Fig 2A). The biomass traits did not identify any significant SNPs either (S5 Fig).

Heatmaps showing *p*-values of the lead SNPs of *qSR8* (8_9225490) and *qSR6-1* (6_7583291) from all of the 217 SR traits indicate that the SNPs had similarly low *p*-values and passed the significant cutoff threshold over a wide range of reflectance ratios (S7 Fig). Such a pattern suggests that the identified SNPs and genetic loci are likely not false positives from noisy data.

Out of 10 *qSR* loci, one of them (*qSR8*) is colocalized with *qPi8-1*. This peak contains three significant SNPs, which are strongly linked (*r*^2^ ≥ 0.9) and located in two genes encoding Suppressor of MAX2-like protein (OsSMAX1, LOC_Os08g15230) and anthocyanidin 3-O-glucosyltransferase (LOC_Os08g15330), which have been shown to be related to Pi deficiency responses. The enzyme anthocyanidin 3-O-glucosyltransferase (EC 2.4.1.115) catalyzes a step in the anthocyanin biosynthesis pathway and its expression has been shown to be up-regulated by Pi deficiency in suspension-cultured grape cells and rice transcriptomic studies [18,19], consistent with Pi starvation-induced anthocyanin accumulation [20,21]. A recent study showed that OsSMAX1 functions in the karrikin signaling pathway downstream of the Dwarf14-Like (D14L) karrikin receptor and negatively regulates arbuscular mycorrhizal (AM) symbiosis and strigolactone biosynthesis [22], which are adaptive strategies to overcome Pi deficiency [23]. These suggest that both Pi contents and SR traits are relevant to each other and to P use efficiency in rice.

The other 9 *qSR* loci are specific to the SR traits. The *qSR6-1* locus includes 30 significant SNPs located in 12 genes (Table 3). Among these genes, *LOC_Os06g13810* encodes the regulatory β-subunit of pyrophosphate-fructose 6-phosphate 1-phosphotransferase (PFP1b). The enzyme PFP (also called PPi-dependent phosphofructokinase, PPi-PFK; EC 2.7.1.90) catalyzes the reversible ‬phosphorylation ‬of ‬fructose ‬6-phosphate to ‬fructose ‬1,6-bisphosphate in the glycolysis pathway and is involved in various stress responses, including phosphate starvation and anoxia in which cytoplasmic Pi and ATP are limited [24,25]. For the *qSR11* locus, a gene encoding an R2R3-type MYB transcription factor OsMYB4P (LOC_Os11g35390), whose overexpression in rice increases Pi acquisition [18], was found in the linkage disequilibrium (LD) block of *qSR11* lead SNP (*r*^2^ = 0.84) (S3 Table).

**Table 3:** List of putative candidate genes associated with Pi content and spectral reflectance indices.

Loci name	Gene ID (MSU)	Gene ID (RAPDB)	Gene annotation
*qSR1-1*	LOC_Os01g19950	-	expressed protein
*qPi1*	LOC_Os01g32890	Os01g0512400	expressed protein
*qSR1-2*	LOC_Os01g66490	Os01g0888300	no apical meristem protein
*qSR2*	LOC_Os02g02370	Os02g0114800	Myb-like DNA-binding domain containing protein
	LOC_Os02g02670	Os02g0118875	NBS-LRR disease resistance protein
	LOC_Os02g02690	Os02g0119100	expressed protein
*qSR3-1*	LOC_Os03g43720	Os03g0638200	transporter family protein
	LOC_Os03g43730	Os03g0638300	tesmin/TSO1-like CXC domain containing protein
*qSR3-2*	LOC_Os03g48230	Os03g0687900	expressed protein
*qPi5*	LOC_Os05g28090	Os05g0348100	expressed protein
*qSR6-1*	LOC_Os06g13650	Os06g0245700	alpha-mannosidase 2
	LOC_Os06g13660	Os06g0245800	alanyl-tRNA synthetase
	LOC_Os06g13670	Os06g0245900	E2F family transcription factor protein
	LOC_Os06g13680	Os06g0246000	B12D protein
	LOC_Os06g13710	Os06g0246400	glycosyltransferase
	LOC_Os06g13730	Os06g0246600	glutamate receptor precursor
	LOC_Os06g13750	-	expressed protein
	LOC_Os06g13760	Os06g0247000	glycosyl transferase 8 domain containing protein
	LOC_Os06g13780	Os06g0247200	expressed protein
	LOC_Os06g13810	Os06g0247500	PFP1, pyrophosphate—fructose 6-phosphate 1-phosphotransferase subunit beta
	LOC_Os06g13820	Os06g0247800	dynamin
*qSR6-2*	LOC_Os06g40740	Os06g0609800	expressed protein
*qSR6-3*	LOC_Os06g44020	Os06g0649100	expressed protein
*qPi8-1*, *qSR8*	LOC_Os08g15230	Os08g0250900	SMAX1-like protein
	LOC_Os08g15330	Os08g0253100	anthocyanidin 3-O-glucosyltransferase
*qPi8-2*	LOC_Os08g29854	Os08g0388300	RGH1A
	LOC_Os08g29870	Os08g0388400	expressed protein
	LOC_Os08g30070	Os08g0390100	expressed protein
*qSR11*	LOC_Os11g35300	-	expressed protein
	LOC_Os11g35320	Os11g0557300	BSD domain-containing protein
	LOC_Os11g35860	Os11g0565300	OsWAK120—OsWAK receptor-like protein kinase
*qPi11*	LOC_Os11g42300	Os11g0642500	OsFBX434—F-box domain containing protein
	LOC_Os11g42900	Os11g0649000	expressed protein

Several loci identified in this study were colocalized with P-related QTLs reported previously [11,26–28] (S8 Fig). Three loci (*qSR3-1*, *qSR3-2*, and *qSR6-2*) were located within the reported marker intervals related to root adaptation traits (e.g. root fresh weight or root number) under P deficiency conditions [27]. These findings suggest that the loci reported in this study likely contribute to P efficiency in rice.

## Discussion

P is an essential macronutrient, and its deficiency leads to limited growth and plant productivity. Recent work showed that P deficiency immediately affects electron transport and CO_2_ assimilation; however, it does not terminate it [29]. Thus, P-deficient plants often do not develop chlorosis and show visual leaf symptoms, especially in young leaves, unless the plants experience long-term severe P deficiency. Consistently, our study showed that Pi contents are more sensitive to plant P status than spectral reflectance. Compared to the P100 group, the P5 group clearly had limited Pi contents, but they did not alter the reflectance spectra. It could be observed in the P0.25 group that P deficiency increased reflectance in the VIS range but reduced reflectance in the NIR range (Fig 1B). Correlation analysis further suggested that reflectance ratios computed from two wavebands, one from NIR (730–790 nm) and the other from green-yellow (530–640 nm) or red edge (700–730 nm), showed good correlation with leaf Pi contents and also had similar correlation patterns with shoot biomass and the computed PUtE (Figs 3A and S3B). The increase of VIS reflectance and the decrease of NIR reflectance in P-deficient leaves are related to the reduction of chlorophyll content and leaf thickness in P-deficient leaves, respectively [30,31]. In addition, our results suggest that the NIR band and red edge region, which is the boundary between chlorophyll absorption of red wavelengths and scattering of NIR wavelengths by the leaf internal structure, are sensitive to Pi deficiency.

The leaf Pi contents at P100 were exceedingly high, whereas the biomass and leaf reflectance spectra did not show much increase from that of the mildly-deficient P5 plants. A previous study has shown that when P levels are over-sufficient, plants can store up to 85% to 95% of total P in the vacuoles [32]. Thus, the measured parameters affected by P-related metabolism and leaf spectral reflectance may not be able to predict the extra amount of Pi stored in the nonmetabolic pool of P-sufficient plants. Consistently, our artificial neural network models trained to classify P deficiency using reflectance data can clearly distinguish P0.25 samples but had trouble with the other two classes whose reflectance spectra are more similar (Fig 2B). Additionally, even though class activation maps indicated that the models put more emphasis on the red (660–690 nm) and the blue (420–500 nm) regions when differentiating the P5 and P100 conditions and also the red edge and NIR (730–790 nm) region when predicting Pi content (Figs 2D and 3D), the reflectance ratios derived from these wavebands are not highly correlated with the Pi content (Fig 3A). Previous studies have shown that several wavebands, including the UV, blue, green, red, red edge and NIR spectrum, can be used to distinguish nitrogen (N)- and P-deficient leaves from the control leaves and that the specific wavebands are dependent on growth stages of the plants [33,34].

Based on the correlation analysis and the number of significant SNPs identified by GWAS, we selected *R*_740_/*R*_560_ and *R*_750_/*R*_700_ as two spectral ratios that are most biologically relevant to Pi deficiency responses. These ratios are sensitive to the change of Pi content when Pi content was less than 0.1 nmol/mm^2^, which is the level found in P0.25 samples, but not when Pi content was higher than 0.2 nmol/mm^2^. Similar patterns have been reported for the relationship between chlorophyll *a* fluorescence and leaf P concentration in barley [35], which are likely due to the extra P stored in the vacuoles [32].

Our model using the two reflectance ratios to predict Pi contents achieved a poor performance with *R*^2^ of 0.20 for both *R*_740_/*R*_560_ and *R*_750_/*R*_700_ at individual plant levels (Figs 3B and S4A). The significant improvement in *R*^2^ at accession levels to 0.52 and 0.69, respectively, suggests that there is considerable variability among individual plants within the same accessions (S4B Fig). Previous studies have shown that Green NDVI (GNDVI, computed from *R*_550_ and *R*_800_) and Red-edge NDVI (RENDVI, computed from *R*_705_ and *R*_750_), which is closed to the *R*_750_/*R*_700_ ratio used here, could estimate leaf chlorophyll (*R*^2^ > 0.8) and nitrogen contents (*R*^2^ > 0.7) in a maize diversity panel grown under different N levels very well [36]. However, the model performance to predict P contents in the same study was poor (*R*^2^ < 0.1) [36]. Although our artificial neural network model achieved a much higher *R*^*2*^ statistics of 0.53 (Fig 3C) by using the information from the whole reflectance spectrum, its performance still lagged behind those reported for leaf chlorophyll and nitrogen content prediction [36]. Due to the latent effect of P deficiency on chlorosis, spectral reflectance may have more limitation in detecting mild levels of P deficiency, compared to N deficiency. On the other hand, a predictive model based on chlorophyll *a* fluorescence transient analysis has been used to predict leaf P concentration with *R*^*2*^ of 0.8 [35]. P deficiency reduces Pi concentration in the chloroplast, inhibits ATP synthase activity and consequently affects the electron transport chain, resulting in the transient change of chlorophyll *a* fluorescence [29]. A recent study developed a model to predict N content of rice using the leaf hyperspectral profile and suggested that collecting reference spectral reflectances and reference N content of rice at various developmental stages would be necessary to accurately evaluate the N status of rice [37]. These findings indicated that several factors, such as plant growth stages and the measured biochemical and physiological parameters, could have a significant impact on the reported *R*^*2*^ statistics in different studies.

Only the Pi content determined from the P5 treatment resulted in significant SNPs, but not the P100 or P0.25 treatments, likely due to oversaturated or extremely scarce Pi contents in these two extreme conditions. Our GWAS of the SR traits identified an overlap set of genes with GWAS of the Pi traits and included more significant SNPs. The identification of genes or QTLs with known P-related functions, such as MYB4P and PFP1b, from the SR-specific traits further supports the potential of other *qSR*-linked candidate genes. Recently, Sun et al. (2019) has shown that the Normalized Difference Spectral Index (NDSI, computed from *R*_1177_ and *R*_1227_) is highly correlated with protein content in rice seeds (*R*^2^ = 0.68). The NDSI trait was also used in GWAS analysis and identified the same SNP loci as rice protein content measured by traditional methods with one extra SNP marker [38].

## Conclusions

These applications of hyperspectral technology in plant phenotyping highlight the advantages of using a non-destructive approach to estimate plant physiological and biochemical traits. In this study, we showed that the hyperspectral technology could be used as a high-throughput phenotyping tool for developing classifier of P deficiency and identifying genetic loci associated with P use efficiency in rice.

## Supporting information

S1 Fig Rice seedlings grown under different P conditions. (a) The experimental setup showing rice seedlings of 172 accessions grown in 80-L containers under P100, P5 and P0.25 conditions. Senescence was visible in the first few leaves of P5 plants. The P0.25 plants showed senescence as well as shoot growth reduction, when compared to the P100 plants. (b) Average root length of ten Thai landrace varieties showing root elongation in response to P deficiency. Error bars indicate standard deviation (*n* = 9).
(TIF)

S2 Fig Hierarchical clustering of variances of mean Pi contents, biomass (shoot dry weight, DW) and P Utilization Efficiency (PUtE) of 172 Thai landrace varieties grown under P100, P5 and P0.25 conditions. (a) Clustering of the traits. (b) Heatmap analysis and clustering of the rice varieties showing different adaptation strategies to limited P supply.
(TIF)

S3 Fig Heatmaps showing Spearman’s correlation. (a) between reflectances at two wavelengths within the same spectrum and (b) between 217 reflectance ratio indices (*R*_NIR_ /*R*_VIS_) and shoot biomass and P Utilization Efficiency (PUtE) computed from shoot biomass divided by Pi content.
(TIF)

S4 Fig Regression analysis between R740/R560 and R750/R700 with Pi contents at individual level (a) and accession level (b-c). The data were fitted by a non-linear regression model with an exponential decay function. The formula and *R*^2^ statistics are displayed in the graph. (a) Each data point is from an individual plant (*n* = 172 accessions x 2 P treatments (P5 and P0.25) x 3 individual plants x 3 independent experiments). (b-c) Each data point is an average value of each rice accession from the same treatment (*n* > 9–12) (*n* = 172 accession x 2 P treatments (P5 and P0.25)).
(TIF)

S5 Fig Additional Manhattan plots from GWAS of leaf Pi content, PUtE, biomass and spectral reflectance. Corresponding P conditions were marked above each plot. The x-axis represents SNP positions across the entire rice genome by chromosome, and the y-axis is the -log_10_ p-value of each SNP.
(TIF)

S6 Fig The number of significant SNPs identified from GWAS of 217 reflectance ratio traits. The ratios with *R*_VIS_ of 420–520 nm yield no significant SNPs and are removed from the plot.
(TIF)

S7 Fig Heatmaps showing -log10(*p*-value) of the lead SNPs of *qSR8* (8_9225490) and *qSR6-1* (6_7583291) from all of the 217 SR traits. (TIF)

S8 Fig Mapping of reported P-related QTL positions and significant candidate genes from this study (listed in the box). P-related QTL intervals and SNPs reported previously are indicated by lines and dots, respectively. The color symbols indicate loci identified from different studies: Li et al., 2009 (red), Ni et al., 1998 (green), Wissuwa et al., 1998 (blue), and Jewel et al., 2019 (yellow).
(TIF)

S1 Table P deficiency classification performances (P100, P5, and P0.25) on the test set. (DOCX)

S2 Table ANOVA analysis for the Pi content, shoot biomass, PUtE and reflectance ratio traits across three P levels in 172 rice accessions. (DOCX)

S3 Table List of LD candidate genes associated with Pi content and spectral reflectance indices. (DOCX)

S1 File List of 172 rice accessions used in this study. (XLSX)

S2 File Pi contents, growth and spectral reflectance data of all rice accessions from different P treatments. The values are means of at least 9 plants per accession per treatment.
(XLSX)
