# Supplementary figures and images for: Hyperspectral and genome-wide association analyses of leaf phosphorus status in local Thai indica rice
Source: PLoS One. 2022 Apr 20;17(4):e0267304. doi: 10.1371/journal.pone.0267304 (PMC9020724; doi:10.1371/journal.pone.0267304)

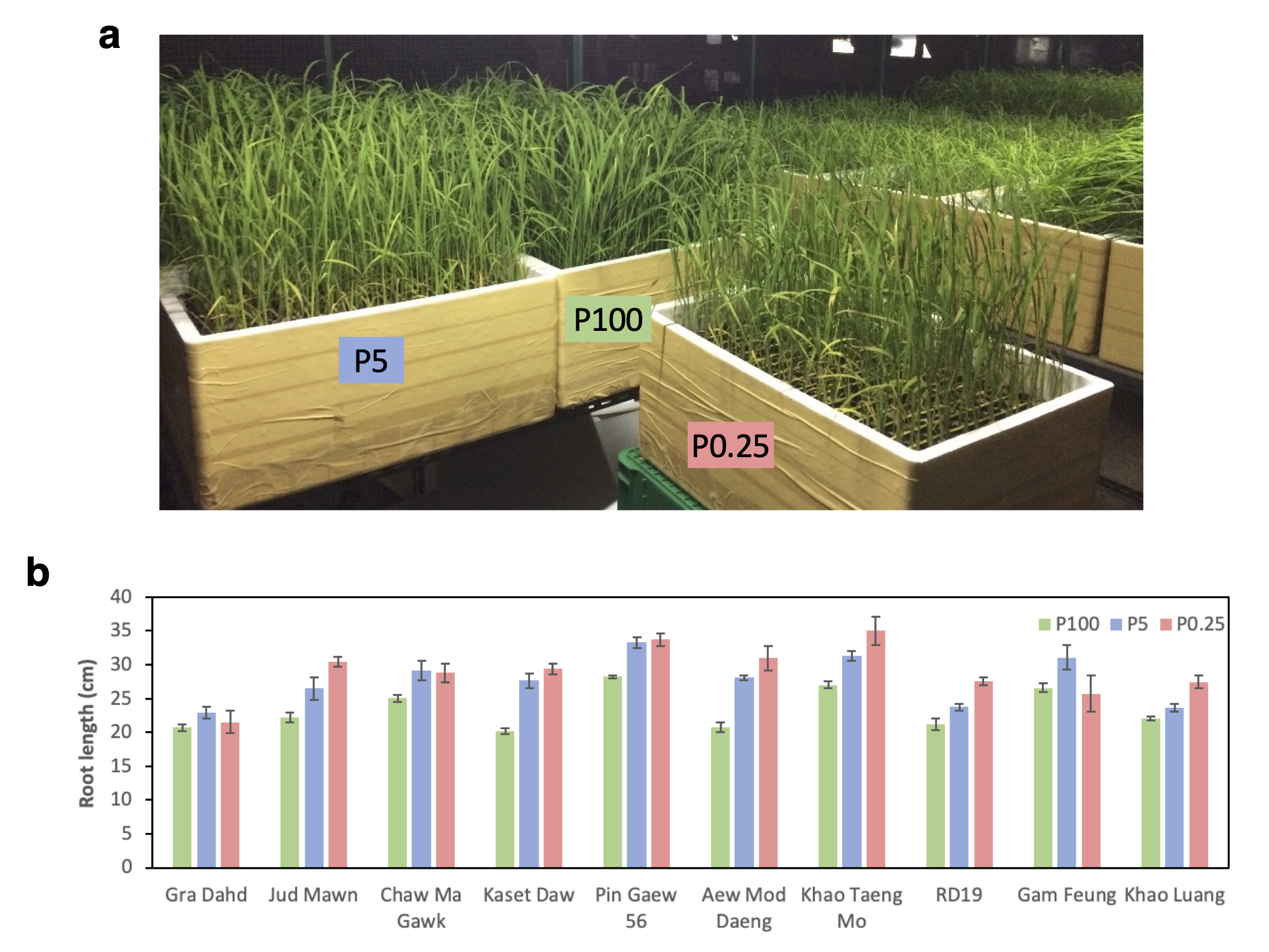

Supplement: S1 Fig — (a) The experimental setup showing rice seedlings of 172 accessions grown in 80-L containers under P100, P5 and P0.25 conditions. Senescence was visible in the first few leaves of P5 plants. The P0.25 plants showed senescence as well as shoot growth reduction, when compared to the P100 plants. (b) Average root length of ten Thai landrace varieties showing root elongation in response to P deficiency. Error bars indicate standard deviation (n = 9). (TIF) [file pone.0267304.s001.tif]

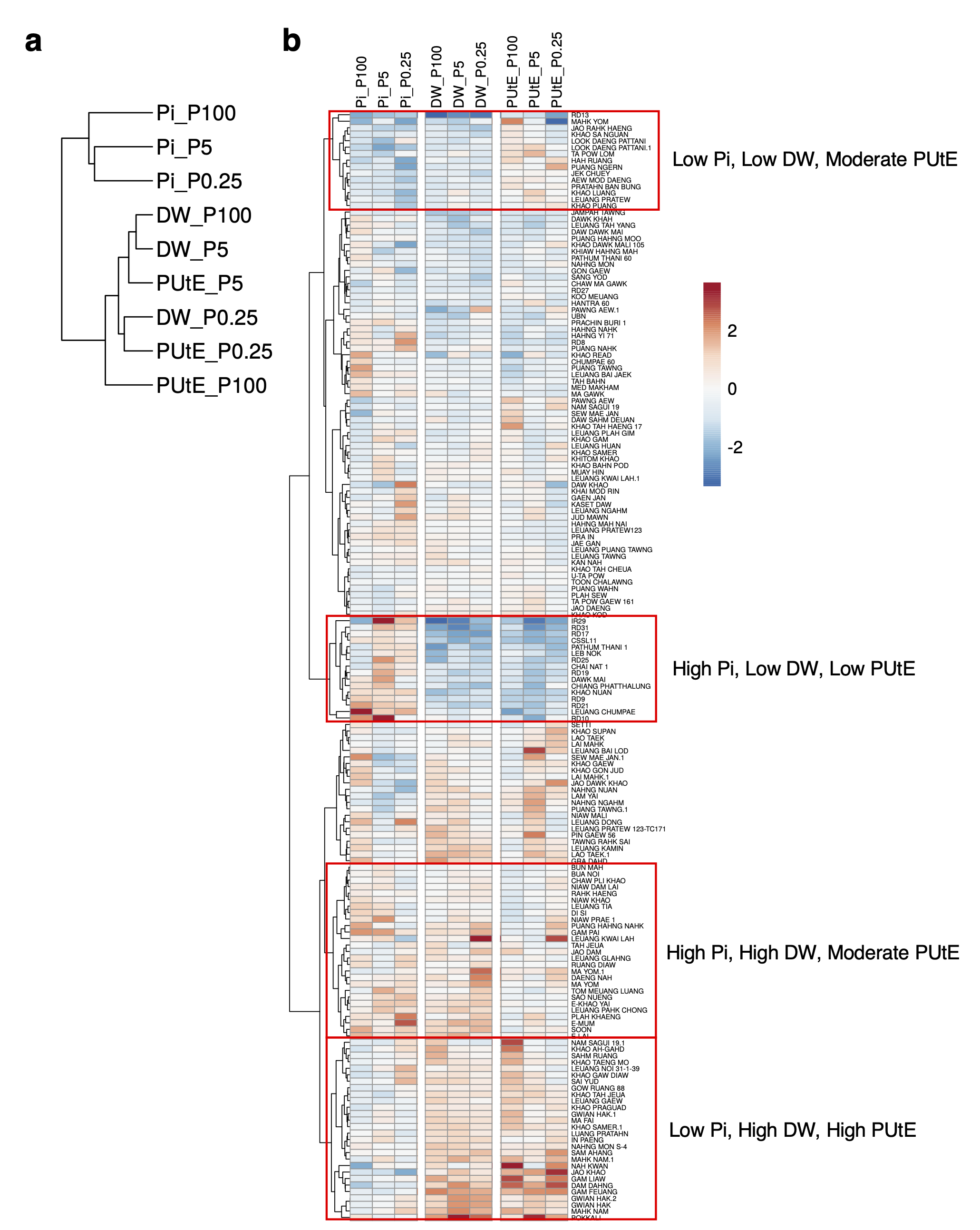

Supplement: S2 Fig — (a) Clustering of the traits. (b) Heatmap analysis and clustering of the rice varieties showing different adaptation strategies to limited P supply. (TIF) [file pone.0267304.s002.tif]

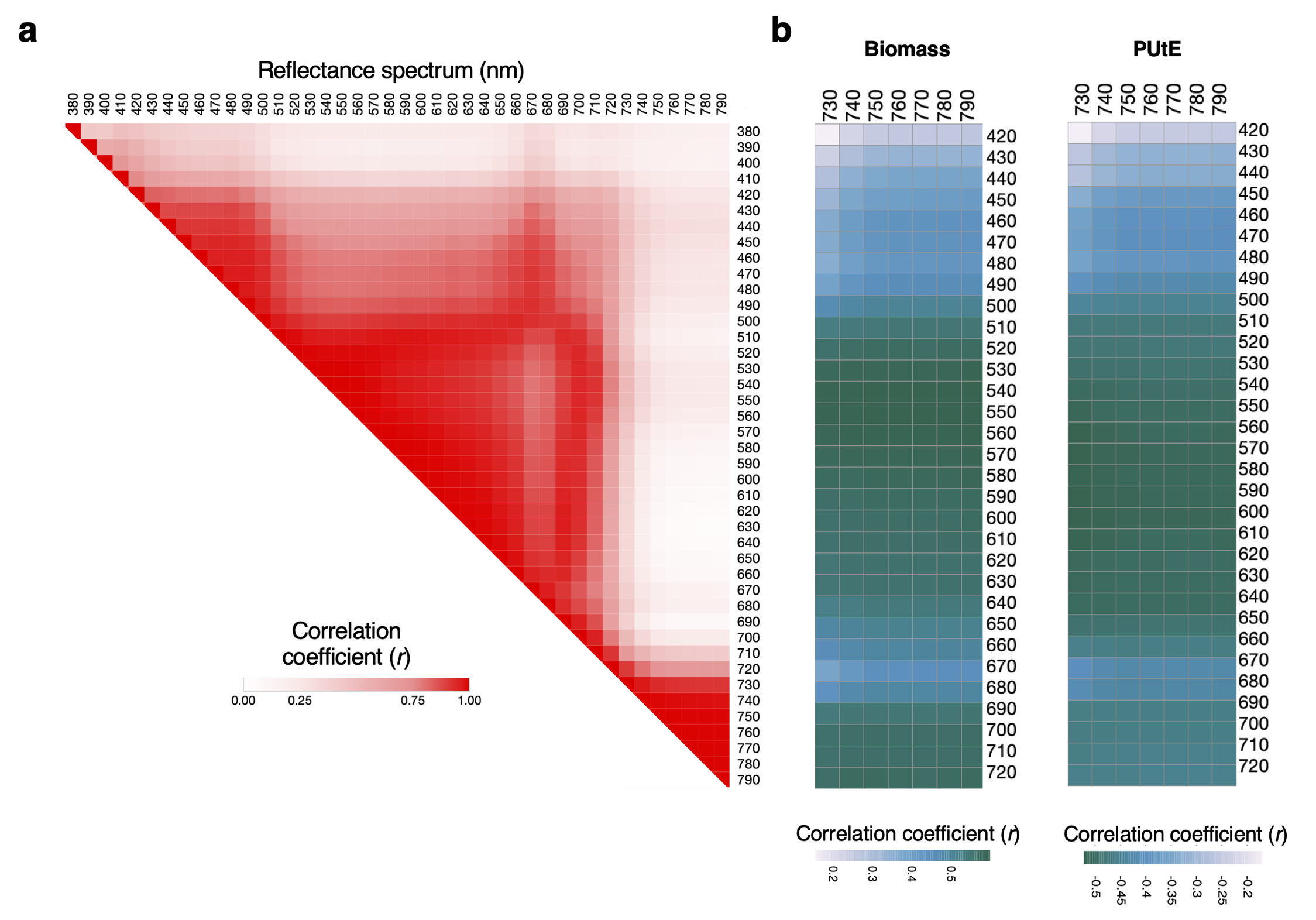

Supplement: S3 Fig — (a) between reflectances at two wavelengths within the same spectrum and (b) between 217 reflectance ratio indices (RNIR /RVIS) and shoot biomass and P Utilization Efficiency (PUtE) computed from shoot biomass divided by Pi content. (TIF) [file pone.0267304.s003.tif]

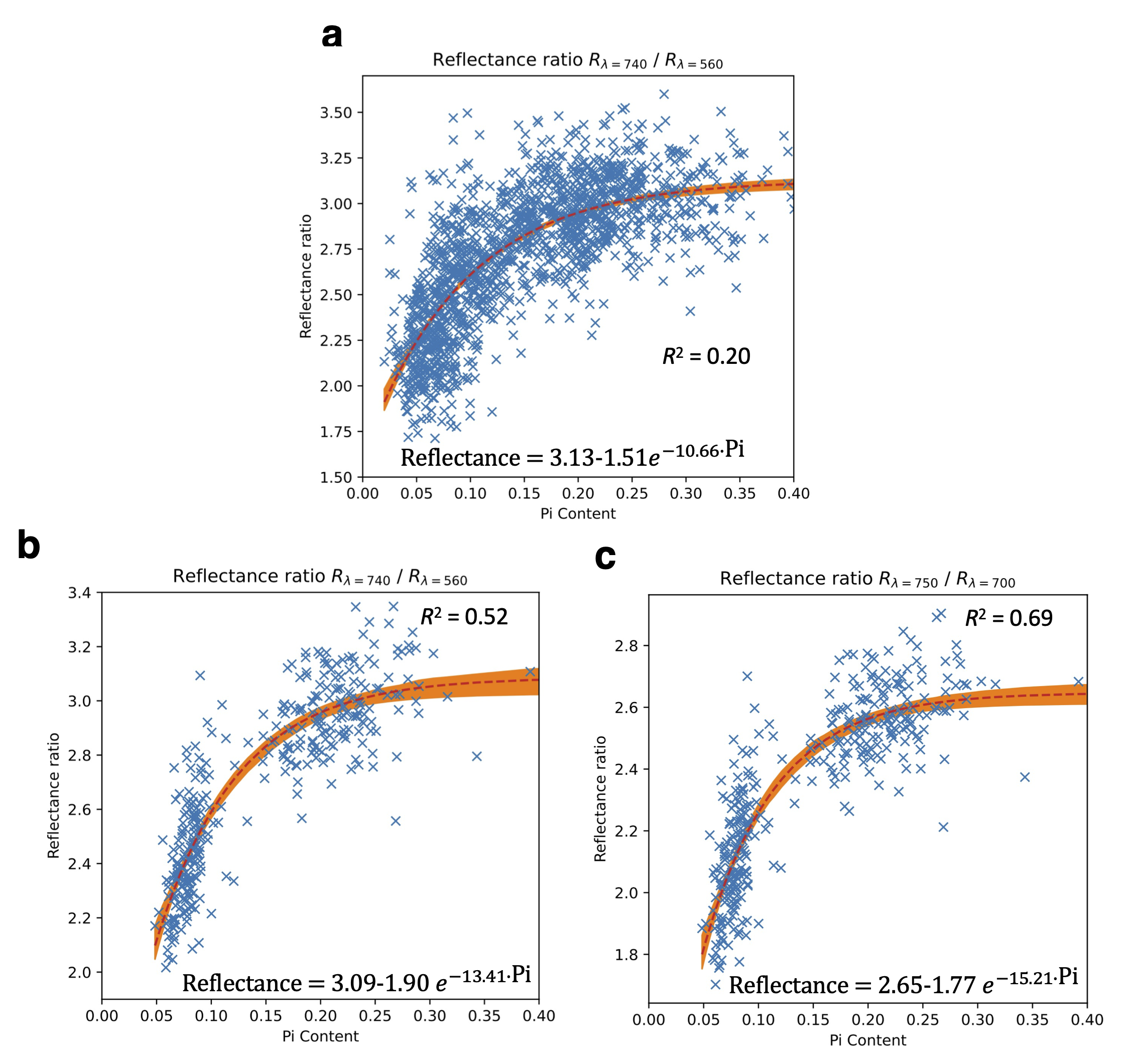

Supplement: S4 Fig — The data were fitted by a non-linear regression model with an exponential decay function. The formula and R2 statistics are displayed in the graph. (a) Each data point is from an individual plant (n = 172 accessions x 2 P treatments (P5 and P0.25) x 3 individual plants x 3 independent experiments). (b-c) Each data point is an average value of each rice accession from the same treatment (n > 9–12) (n = 172 accession x 2 P treatments (P5 and P0.25)). (TIF) [file pone.0267304.s004.tif]

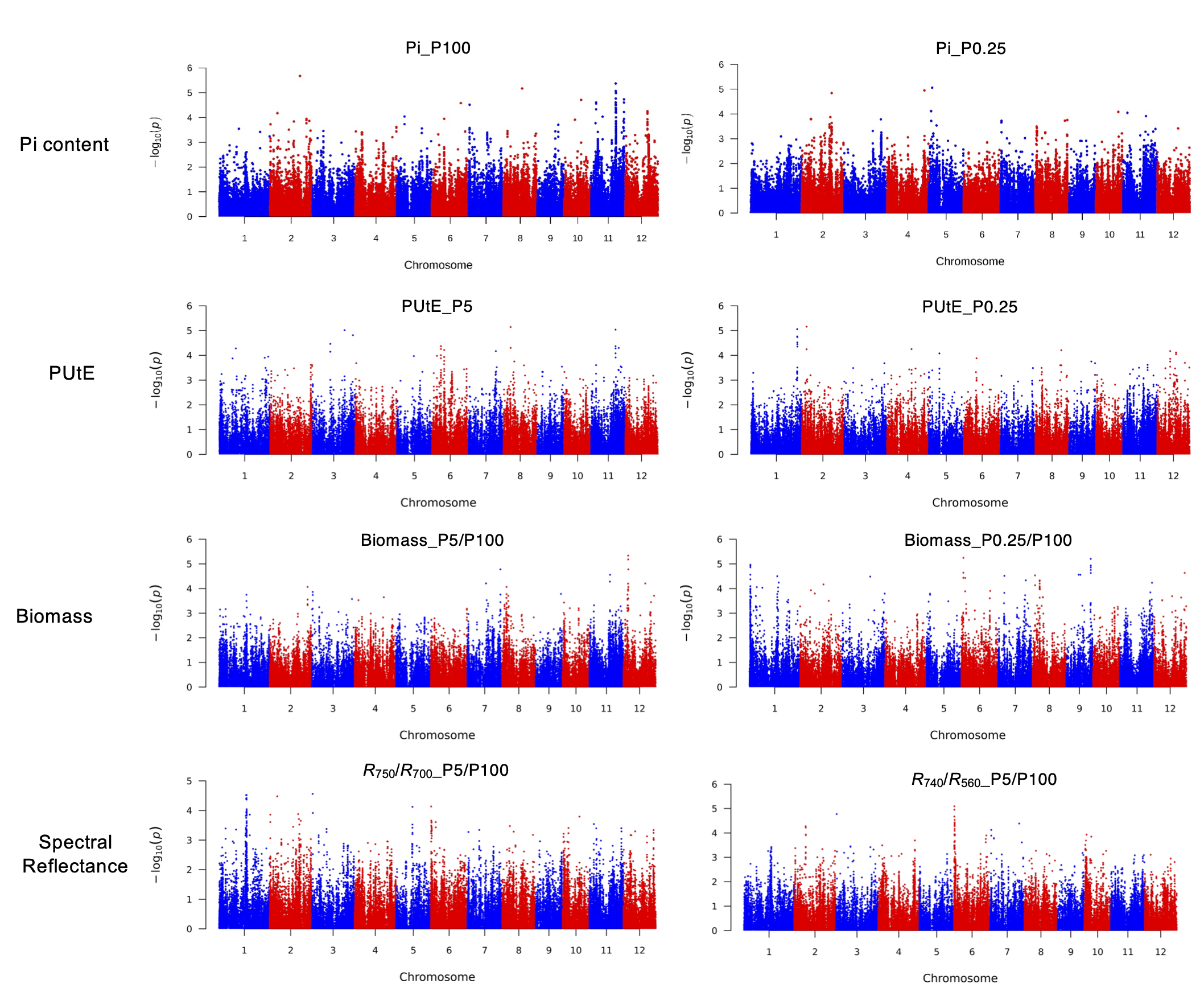

Supplement: S5 Fig — Corresponding P conditions were marked above each plot. The x-axis represents SNP positions across the entire rice genome by chromosome, and the y-axis is the -log10 p-value of each SNP. (TIF) [file pone.0267304.s005.tif]

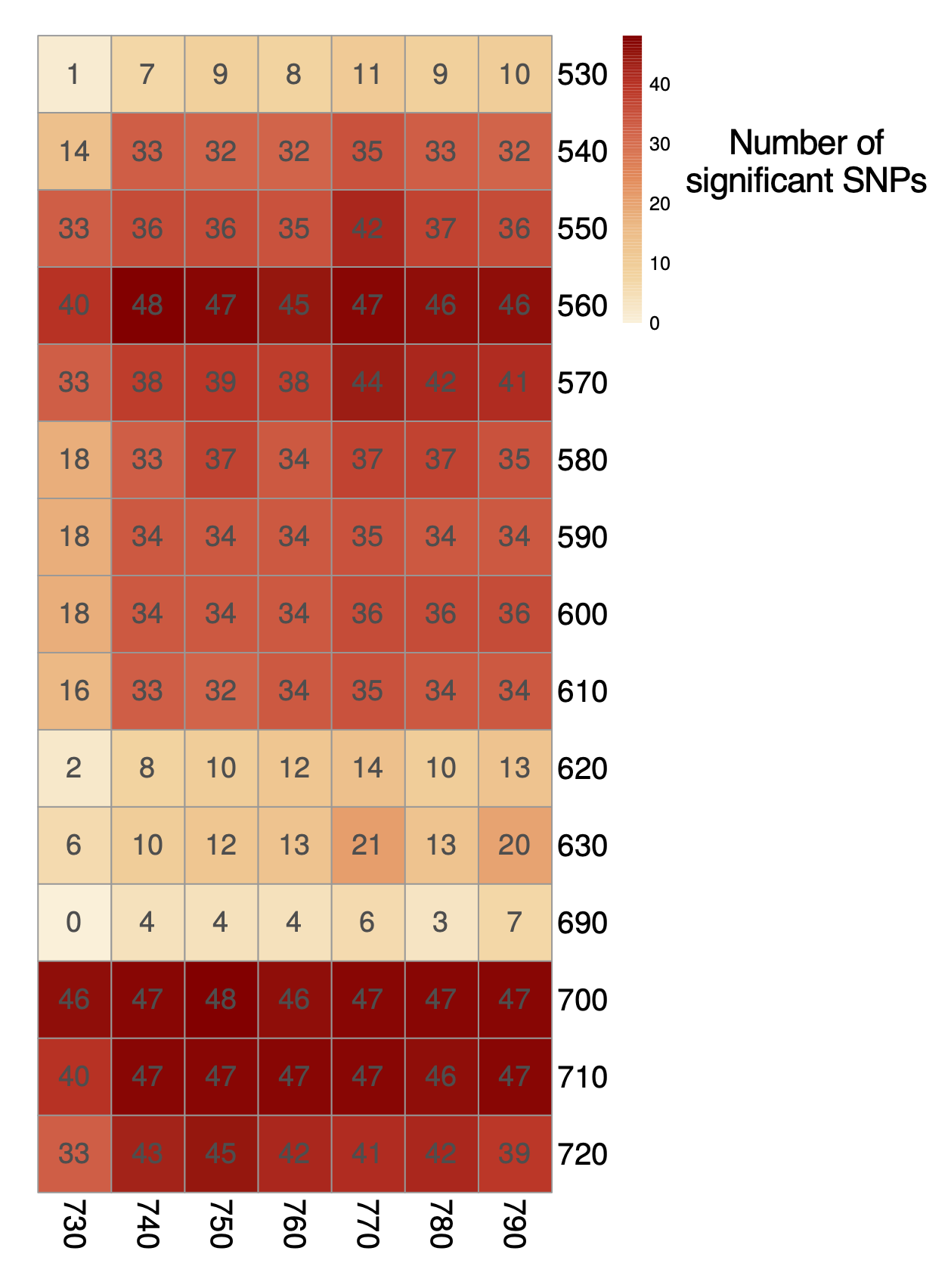

Supplement: S6 Fig — The ratios with RVIS of 420–520 nm yield no significant SNPs and are removed from the plot. (TIF) [file pone.0267304.s006.tif]

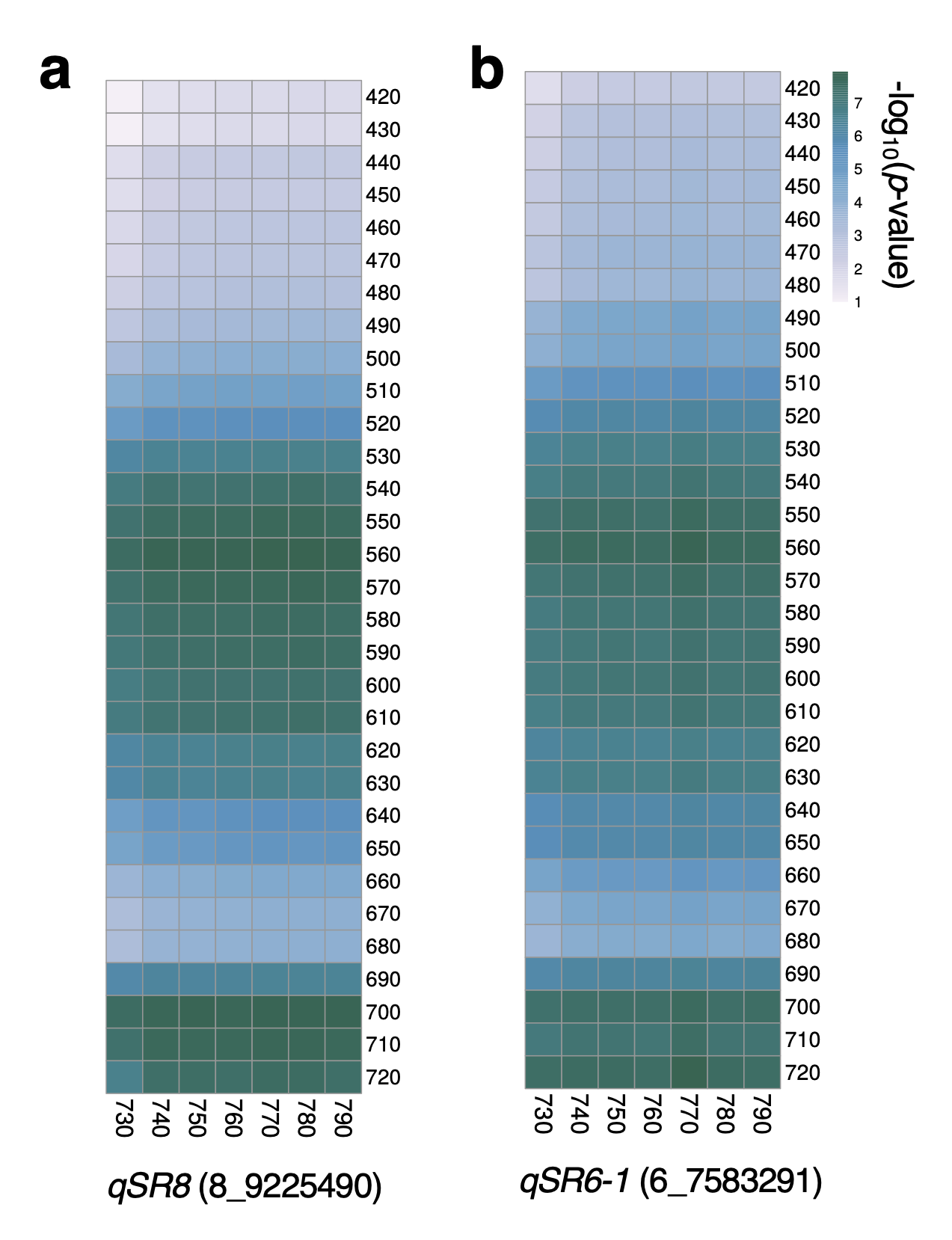

Supplement: S7 Fig — (TIF) [file pone.0267304.s007.tif]

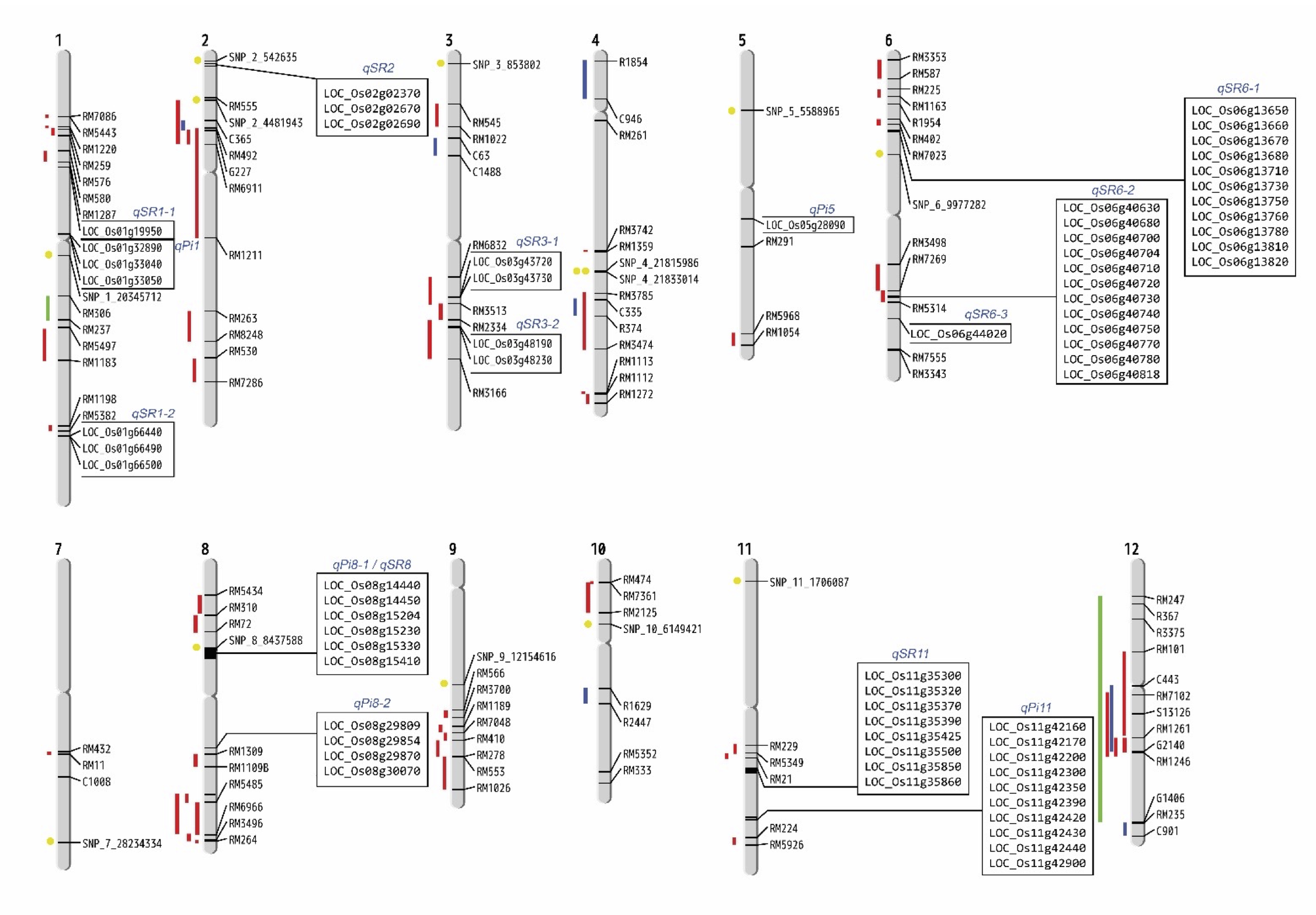

Supplement: S8 Fig — P-related QTL intervals and SNPs reported previously are indicated by lines and dots, respectively. The color symbols indicate loci identified from different studies: Li et al., 2009 (red), Ni et al., 1998 (green), Wissuwa et al., 1998 (blue), and Jewel et al., 2019 (yellow). (TIF) [file pone.0267304.s008.tif]
