# Supplementary material for: Hyperspectral and genome-wide association analyses of leaf phosphorus status in local Thai indica rice
Source: PLoS One. 2022 Apr 20;17(4):e0267304. doi: 10.1371/journal.pone.0267304 (PMC9020724; doi:10.1371/journal.pone.0267304)
Supplement: S1 Table — (DOCX) [file pone.0267304.s009.docx]

**S1 Table.** P deficiency classification performances (P100, P5, and P0.25) on the test set.

| **Model** | **Hidden**  **Dimension** | **Accuracy**  **(Mean** $\boldsymbol{\pm}$ **SD)** |
| --- | --- | --- |
| Classification | 32 | 0.8475 $\pm$ 0.0042 |
|  | 64 | 0.8521 $\pm$ 0.0053 |
|  | 128 | 0.8460 $\pm$ 0.0053 |
|  | 256 | 0.8443 $\pm$ 0.0049 |
|  | 512 | 0.8458 $\pm$ 0.0051 |
|  | 1024 | 0.8440 $\pm$ 0.0027 |
| Multitask | 32 | 0.8506 $\pm$ 0.0028 |
|  | 64 | 0.8560 $\pm$ 0.0030 |
|  | 128 | 0.8513 $\pm$ 0.0039 |
|  | 256 | 0.8474 $\pm$ 0.0029 |
|  | 512 | 0.8481 $\pm$ 0.0022 |
|  | 1024 | 0.8445 $\pm$ 0.0067 |
