# Supplementary material for: Hyperspectral and genome-wide association analyses of leaf phosphorus status in local Thai indica rice
Source: PLoS One. 2022 Apr 20;17(4):e0267304. doi: 10.1371/journal.pone.0267304 (PMC9020724; doi:10.1371/journal.pone.0267304)
Supplement: S2 Table — (DOCX) [file pone.0267304.s010.docx]

**S2 Table.** ANOVA analysis for the Pi content, shoot biomass, PUtE and reflectance ratio traits across three P levels in 172 rice accessions.

| Trait | Genotype | Treatment | Genotype  x Treatment | Error |
| --- | --- | --- | --- | --- |
| Pi content | 1.92* | 5720.43* | 1.84* | 0.74 |
| Shoot biomass | 0.05* | 9.40* | 0.007* | 0.003 |
| PUtE | 0.20* | 220.09* | 0.11* | 0.08 |
| *R*﻿_740_/*R*﻿_560_ | 0.39* | 149.52* | 0.09* | 0.01 |
| *R*﻿_750_/*R*﻿_700_ | 0.28* | 104.13* | 0.08* | 0.02 |

The numbers indicate the mean square value. Significant level at *p* < 0.0001 was indicated by *.
