# Supplementary material for: Hyperspectral and genome-wide association analyses of leaf phosphorus status in local Thai indica rice
Source: PLoS One. 2022 Apr 20;17(4):e0267304. doi: 10.1371/journal.pone.0267304 (PMC9020724; doi:10.1371/journal.pone.0267304)
Supplement: S3 Table — (DOCX) [file pone.0267304.s011.docx]

**S3 Table.** List of LD candidate genes associated with Pi content and spectral reflectance indices.

| Loci name | Gene ID  (MSU) | Gene ID (RAPDB) | *r*^2^ | Gene annotation |
| --- | --- | --- | --- | --- |
| *qPi1* | LOC_Os01g33040 | Os01g0513900 | 0.65 | kinesin motor domain containing protein |
|  | LOC_Os01g33050 | Os01g0514000 | 0.65 | ribosomal protein L24 |
| *qSR1-2* | LOC_Os01g66500 | Os01g0888500 | 0.85 | phosphoribosylformylglycinamidine synthase |
| *qSR3-2* | LOC_Os03g48190 | Os03g0687200 | 0.92 | expressed protein |
| *qSR6-1* | LOC_Os06g13860 | Os06g0248400 | 0.55 | RCD1 |
| *qSR6-2* | LOC_Os06g40630 | Os06g0608600 | 0.55 | SFT2 |
|  | LOC_Os06g40680 | - | 0.58 | expressed protein |
|  | LOC_Os06g40700 | Os06g0609301 | 0.64 | OsSub50 - Putative Subtilisin homologue |
|  | LOC_Os06g40704 | Os06g0609450 | 0.64 | stromal membrane-associated protein |
|  | LOC_Os06g40710 | Os06g0609500 | 0.62 | Myb-like DNA-binding domain containing protein |
|  | LOC_Os06g40720 | Os06g0609600 | 0.61 | EF hand family protein |
|  | LOC_Os06g40730 | Os06g0609700 | 0.62 | protein with a conserved N-terminal region |
|  | LOC_Os06g40750 | Os06g0609900 | 0.98 | expressed protein |
|  | LOC_Os06g40770 | Os06g0610100 | 0.98 | expressed protein |
| *qPi8-1, qSR8* | LOC_Os08g14440 | Os08g0242700 | 0.50 | uridylyltransferase-related |
|  | LOC_Os08g14450 | Os08g0242800 | 0.66 | RNA polymerase sigma factor |
|  | LOC_Os08g15204 | Os08g0250700 | 0.83 | thioredoxin domain-containing protein 9 |
|  | LOC_Os08g15410 | - | 0.86 | expressed protein |
| *qPi8-2* | LOC_Os08g29809 | Os08g0387700 | 0.62 | resistance protein LR10, putative, expressed |
| *qSR11* | LOC_Os11g35370 | Os11g0558000 | 0.79 | expressed protein |
|  | LOC_Os11g35390 | Os11g0558200 | 0.84 | MYB family transcription factor |
|  | LOC_Os11g35425 | - | 0.76 | expressed protein |
|  | LOC_Os11g35500 | Os11g0559200 | 0.57 | receptor-like protein kinase 5 precursor |
|  | LOC_Os11g35850 | Os11g0565000 | 0.92 | expressed protein |
| *qPi11* | LOC_Os11g42160 | Os11g0641200 | 0.76 | F-box/LRR-repeat protein 3 |
|  | LOC_Os11g42170 | Os11g0641300 | 0.79 | expressed protein |
|  | LOC_Os11g42200 | Os11g0641500 | 0.98 | laccase precursor protein |
|  | LOC_Os11g42350 | Os11g0642800 | 0.98 | glutathione synthetase, chloroplast precursor |
|  | LOC_Os11g42390 | Os11g0643400 | 0.97 | OsSCP64 - Putative Serine Carboxypeptidase homologue |
|  | LOC_Os11g42420 | Os11g0643700 | 0.91 | nuclear pore protein 84/107 containing protein |
|  | LOC_Os11g42430 | Os11g0643800 | 0.63 | transporter family protein |
|  | LOC_Os11g42440 | Os11g0644000 | 0.76 | expressed protein |
